# Differential editing efficiencies in cereal crops: a comparative analysis of tRNA and ribozyme multiplexed guide delivery

**DOI:** 10.3389/fpls.2024.1426184

**Published:** 2024-12-05

**Authors:** Matthew J. Milner, Manisha Sharma, Ruth E. Bates, Michelle Whiting, Melanie S. Craze, Peter Miller, Jack Brooks, Allan Kouidri, Emma J. Wallington

**Affiliations:** NIAB, Cambridge, United Kingdom

**Keywords:** CRISPR, tRNA, ribozyme, rice, wheat, barley, Gsk1, CmYLCV

## Abstract

Cereal transformation and gene editing can be a complex and costly undertaking. It is therefore important to validate and understand the performance of the components to achieve high rates of transformation and gene editing. Here, we have made a direct comparison of different CRISPR/Cas9 guide systems to target the genome in three cereal species. We show that the guide sequences driven by the same pol II promoter in rice, wheat and barley show large differences in editing efficiency. The differences seen were based on the way the guides were presented and factors outside of the guide sequence itself. While both the tRNA system and ribozyme system performed well in rice, their effectiveness varied in wheat and barley. Specifically, the tRNA system outperformed the ribozyme system, achieving higher rates of editing in stable transformed plants. Overall, high levels of editing are observed in all three species when strong expression of the SpCas9 is coupled with the CmYLCV promoter to drive a tRNA array of guide RNAs. Stable inheritance is also achievable in all three species when plants are sampled shortly after the tissue culture concludes. Overall, inheritance rates were above 85% in all three species, particularly when mutations are detected early after plants emerge from tissue culture.

## Introduction

The tools available to successfully modify genomes are continually improving with innovative and novel targeted modifications becoming possible. An understanding of the factors which influence gene editing in different species is paramount for successful and efficient editing of the desired loci. With the advent of CRISPR/Cas9, precision genome editing has become almost routine in many species. Over the past ten years, CRISPR/Cas9 has become widely adopted due to its simplicity of design, ease of use and broad applicability across all kingdoms. Researchers have therefore wanted to increase the number of targets which can be edited simultaneously in a single transformation, but how best to target and deliver the multiple guides is not necessarily clear (Li et al., 2021). As the technology matures researchers are finding that not all guide RNAs cause mutations at the same efficiency, or indeed at all (Feng et al., 2013; Hahn et al., 2020; Howells et al., 2018; Ma et al., 2015; Mikami et al., 2015; Milner et al., 2020a; Naim et al., 2020; Okada et al., 2019; Wang et al., 2014, 2018; Xie and Yang, 2013). Promoter strength and epigenetic factors such as chromatin state have been suggested for the variability observed in editing efficiency (Weiss et al., 2022).

The differences observed in editing, heritability and ploidy in certain plant species has led some to label certain species as problematic (Lawrenson et al., 2015; Ma et al., 2015; Gasparis et al., 2018; LeBlanc et al., 2018; Milner et al., 2020b). In many instances, in the literature supporting the “best” system to use, conclusions are made regarding the performance of components without a direct comparison of the same promoters driving expression of the same guide sequence in stable transformed plants or, within different species (Li et al., 2021). Often these involve the use of a guide selection prediction program, which provides a poor correlation with the actual data determined from stable transformed plants (Milner et al., 2020b; Naim et al., 2020). Thus, a need to understand why certain guide sequences do not cause mutations is important, as more sophisticated targeted edits using base editing or prime editing are now possible (Ren et al., 2018; Lin et al., 2020). To further understand whether guide sequence *per se* or other factors influence the efficiency of editing in cereals we compared two guide delivery systems which allow multiple guides to be used to simultaneously target multiple loci within different cereal species.

Previous work in rice, wheat and barley has shown both tRNA and ribozyme systems to be effective in editing desired locations (Xie et al., 2015; Tang et al., 2016; Čermák et al., 2017; Gasparis et al., 2018; Wang et al., 2018a; Li et al., 2021; Ni et al., 2023). A small number of promoters have been used to drive the expression of the CRISPR guides primarily Pol III type promoters from various species including rice, wheat, and Arabidopsis, or Pol II type viral promoters such as CmYLCV (Čermák et al., 2017; Li et al., 2021). The various promoters and the guide delivery systems have therefore been validated independently in different plant species to create the desired edits.

To understand how the two different guide delivery systems perform in three cereal species we chose a conserved gene involved in the regulation of the brassinosteroid pathway, GSK1. GSK1 is a highly conserved gene that has been shown to be involved in various abiotic stress tolerance in both rice and barley but remains uncharacterized in wheat (Koh et al., 2007; Kloc et al., 2020). By using guides that target the same DNA sequences in three different cereal species, this study shows that the guide delivery system chosen can have a profound effect on the editing outcome as well as the overall ability to edit multiple loci in a polyploid species such as wheat. Overall, while both the tRNA system and ribozyme system work equally well in rice, the tRNA system delivers much better editing outcomes in wheat and barley.

## Methods

### Sequence comparison

The rice protein sequences were taken from (Koh et al., 2007) and used to identify the gene as Os01g0205700 or LOC_Os01g10840 using RAP-DB (https://rapdb.dna.affrc.go.jp/). Additional DNA and amino acid sequences were downloaded from RAP-DB. Wheat GSK1 DNA or protein sequences were obtained from GrainGenes (https://graingenes.org/GG3/) using the rice ortholog protein sequence to search for the three wheat orthologs. These loci using RefSeq2.1 are TraesCS3A03G0312800, TraesCS3B03G0368500LC, and TraesCS3D03G0288900. For the barley GSK1 sequences Ensembl plants (https://plants.ensembl.org/) was used to identify the closest gene in barley, HORVU.MOREX.r3.3HG0243150. Alignment of the DNA and amino acid sequences was performed using Mega X (Kumar et al., 2018).

### Construct design

**Figure 1:**
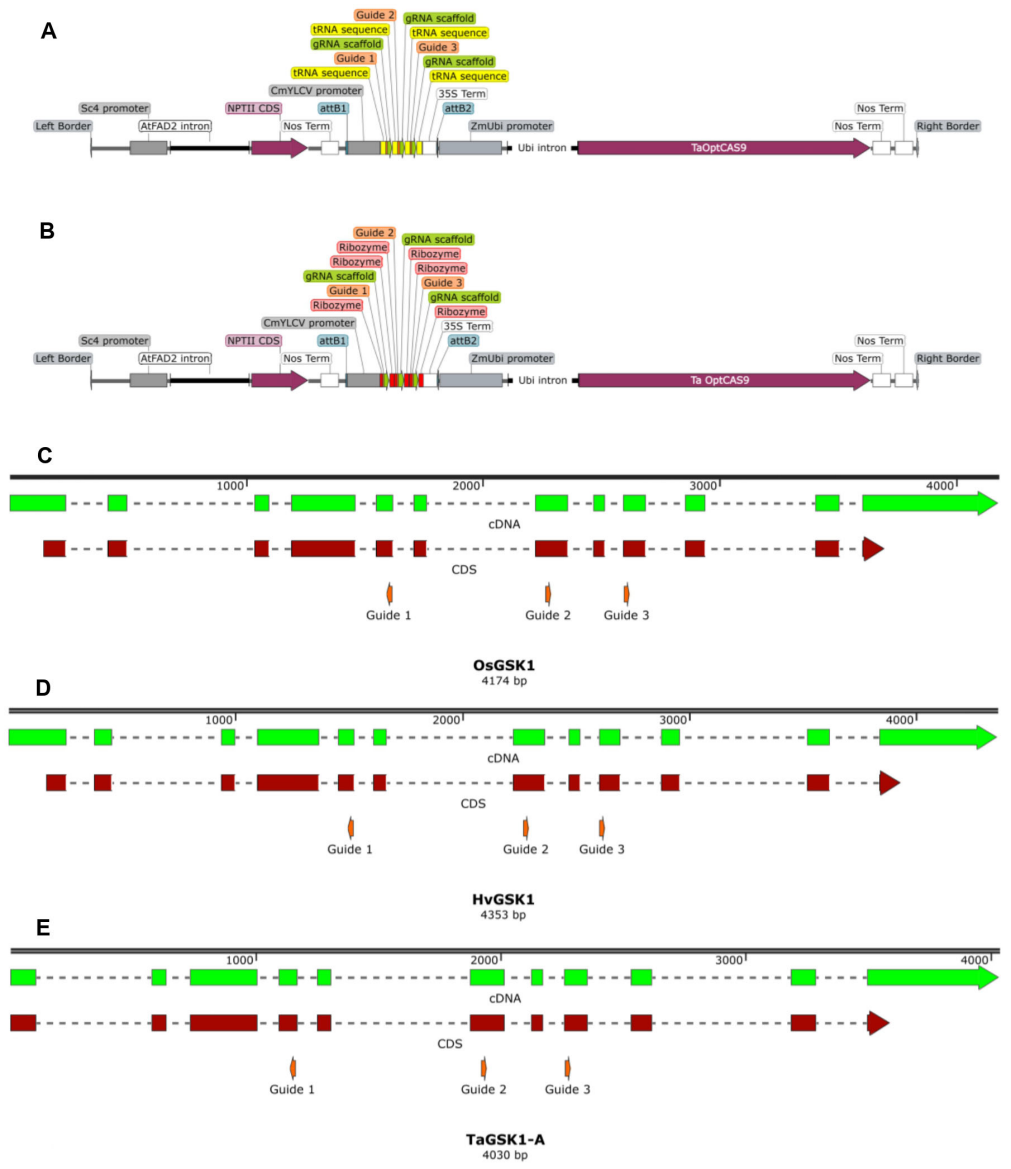
Schematic diagram of the pMM36 **(A)** and pMM37 **(B)** T-DNAs plus the gsk1 genomic sequences from rice **(C)**, barley **(D)** and wheat (A-homoeologue) **(E)** with the guide target sites shown. Distances between target guide PAM sites in rice are 698bp (guides 1-2), 330bp (guides 2-3); in barley 796bp (guides 1-2), 336bp (guides 2-3); and in wheat A-genome 806bp (guides 1-2), 341bp (guides 2-3).

**Figure 2:**
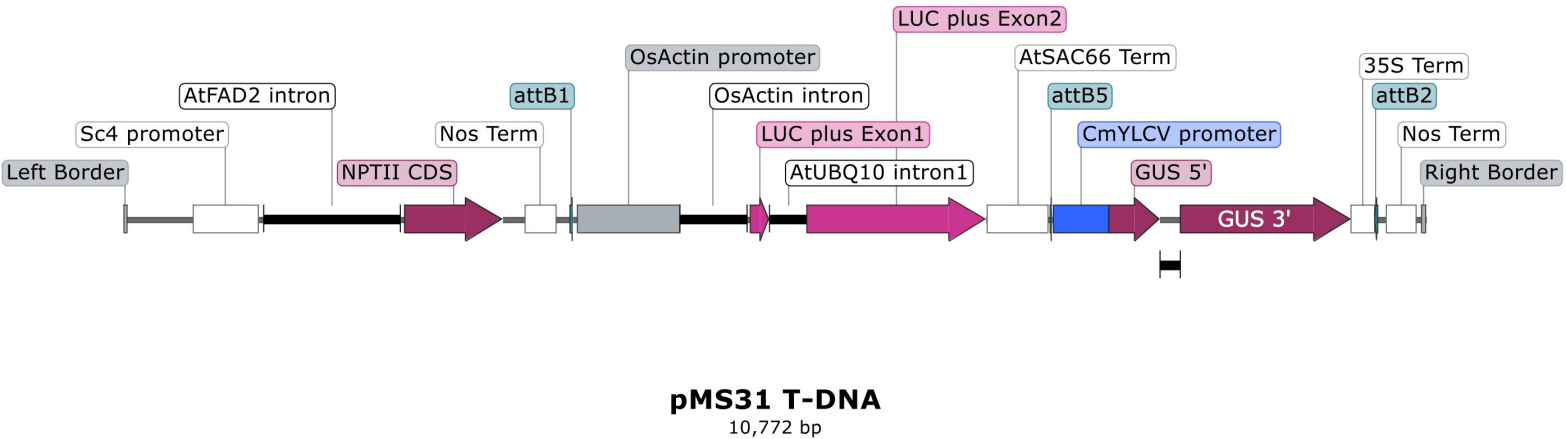
Schematic diagram of promoter construct T-DNA structure. The T-DNA structure for the dual reporter binary construct pMS31 containing the CmYLCV promoter is shown. This includes the OsActin promoter-luciferase cassette and the Sc4 promoter-nptII selection gene cassette. Other promoters were substituted for CmYLCV, namely the TaU6 promoter in pMS33 and the Zea mays Ubiquitin promoter in pAK90 and pAK93 (with hygromycin cassette).

Guides were chosen based on alignments of guides identified from the CRISPR-P 2.0 website (Liu et al., 2017). Three guides were selected which targeted identical sequences in rice, wheat and barley: guide 1 ^5’-^TTTGTGGTTTCACATCCCTGTGG ^-’3^; guide 2 ^5’-^CGTGCTCCTGAGCTCATATTTGG ^-’3^ and guide 3 ^5’-^TCTTGGTACTCCAACCCGTGAGG ^-’3^ where the PAM feature is underlined. The guide stacks were synthesized (Genewiz) using the sequences obtained for either the tRNA or ribozyme systems from the Čermák et al., 2017 with attL1/2 sites added for gateway recombination. Each guide stack was recombined into pEW474-Cas-R1R2 which contains a wheat codon optimized *ScCas9* expressed from the ZmUbi promoter *in planta* to create pMM36 and pMM37, containing the guide stack as a tRNA or ribozyme system respectively (**Figure 1**). Three promoter-GUS reporter constructs, pCmYLCV: GUS, pTaU6:GUS, and pZmUbi: GUS plus a constitutively expressed Luciferase construct, pOsActin: Luc, were created using Goldengate assembly (Engler et al., 2014). Each GUS cassette was then recombined with the pOsActin: Luc cassette into pRLF12-R1R2 or pEW343-R1R2 in a 2-insert multisite cloning reaction with LR Clonase II Plus (Thermo Fisher Scientific), to create pMS31, pMS33, pAK90 and pAK93 (**Figure 2**). All constructs were verified by restriction digest and sequencing. Final binary constructs were transformed into *A. tumefaciens*. The plasmids were then isolated from the Agrobacterium cultures and subjected to additional restriction digest verification before their use in wheat, barley and rice experiments (Bates et al., 2017). A summary of constructs and transformation experiments is provided in **Supplementary Table 1**.

### Rice transformation

Rice cv Nipponbare was transformed essentially as in Roth et al., 2018. G418 (100mg/l) was used as an alternative selective agent to hygromycin. All callusing/transition stages of rice tissue culture were undertaken at 30°C in the dark as standard. Rooted plants were transferred to Jiffy pellets, hardened off and potted in 9cm pots containing M2 compost plus 5g/l slow release fertilizer (Osmocote exact 15:10:10). Plants were grown to maturity and seed harvest in a Conviron chamber (500 μmol/m^-2^ s^-1^, 28°C day/25°C night, 12-hr photoperiod).

### Wheat transformation

Agrobacterium from an overnight plate was resuspended in inoculation medium (Murishige and Skoog salts and vitamins, 0.47g/l, glucose, 10g/l, MES 0.5g with 140µM acetosyringone) to an OD_660_ of approximately 1.0. After surface sterilization of immature caryopses in 10% bleach (containing 5% sodium hypochlorite) with 20µl/50ml Tween 20, embryos were isolated aseptically, at a stage of semi-translucence (14-16 DAA, approximately 1-2mm) into inoculation medium, as above. Inoculation medium was subsequently removed and replaced with 2ml prepared *Agrobacterium* suspension in a sterile 7ml Bijou tube. Open tubes were placed in a sterile vacuum desiccator and a vacuum of -0.08MPa applied for 5 minutes. After vacuum-release, *Agrobacterium* was removed and the embryos co-cultivated for 2-3 days on CO1 medium, (derived from Ishida et al., 2015, containing Murishige and Skoog salts and vitamins 0.47g/l, glucose 10g/l, MES 0.5g/l, CuSO_4_ 1.25mg/l, Sigma Type I agarose 8g/l, 5µM AgNO_3_ and 200µM acetosyringone, pH 5.8). Post co-cultivation, embryonic axes were removed, and the scutella transferred to W4 medium for 2 weeks, followed by the application of selection, essentially as in the SIM protocol of Risacher et al., 2009. Tissue culture plates from experiments with gene editing constructs, were maintained at 28.5°C day/23.5°C night temperature, 16-hr daylength throughout the callus phase until regeneration (Milner et al., 2020b). Otherwise, all tissue culture and transgenic plant growth steps followed those from the SIM protocol except that 2mg/l zeatin was included in the regeneration medium in place of kinetin. 25mg/l G418 was used for selection of the *nptII* gene throughout the callus and regeneration stages of the tissue culture. Regenerated plants with good root development were transferred to Jiffy 7 peat pellets and acclimatized in a propagator, before transfer to 9cm pots as above. Plants were grown to maturity in a Conviron chamber (450-500 μMol m^-2^ s^-1^, 20°C day/15°C night, 16-hr photoperiod).

### Barley transformation

Barley cv. Golden promise was transformed essentially as in Bartlett et al., 2008, except that stock plants for immature embryo production were grown at 18°C day/13°C night, 16-hour daylength, and G418 (50mg/l) was used as a selective agent throughout instead of hygromycin. Additionally, the 6 weeks of culture on callusing medium after co-cultivation was carried out in the dark in a Sanyo growth chamber set at 28.5°C day/23.5 night temperature, 16-hr daylength for experiments with gene editing constructs. Rooted plants were transferred to Jiffy pellets and then 9cm pots as above and grown to maturity in a Conviron chamber (450-500 μMol m^-2^ s^-1^, 18°C day/13°C night, 16-hr photoperiod).

### DNA analysis of transformed plants

DNA from transgenic lines was extracted using crude DNA extraction buffer (200 mM Tris pH 7.5, 250 mM NaCl, 25 mM EDTA, 0.5% SDS), incubated at 65°C for 1 hr then centrifuged at 6000 g for 10 min. The DNA was precipitated by addition of 400 μl propan-2-ol to the supernatant followed by centrifugation, as previously (Howells et al., 2018). DNA pellets were resuspended in 100 μl TE, incubated at 65°C for 5 min and centrifuged at 6000 g for 5 min. DNA was diluted 1:3 in sterile H_2_O prior to use in all assays. T-DNA copy number was determined using a TaqMan relative quantification (ΔΔCT) assay comparing the relative values of a nptII amplicon to an amplicon of the single copy wheat gene SPS for rice, Con2 for Barley and GaMyb for wheat within a multiplexed reaction and normalized to a single copy control (Ding et al., 2004; Yang et al., 2005; Bartlett et al., 2008; Milner et al., 2018) (**Supplementary Table 2**). Primers and Taqman probes were used at a concentration of 200nM in a 10 μl multiplex reaction with ABsolute Blue qPCR ROX mix (Thermo Fisher Scientific Inc.) using the standard run conditions (50°C- 2 min; 95°C-10 min; 40 cycles of 95°C-15 sec, 60°C-1 min) for the ABI 7900 HT (Thermo Fisher Scientific Inc.) for wheat and barley. Modified primer concentrations (50 nM each SPS-F/SPS-R primer and 25 nM SPS-P probe plus 25 nM each Npt2B2F/Npt2B4R primer and 12.5 nM Npt2B2P probe) and run conditions (50°C-2 min; 95°C-15 sec; 40 cycles of 95°C-15 sec, 56°C-30 sec, 60°C-30 sec) were used for rice.

The relative quantification, ΔΔCt, values were calculated to determine nptII copy number in the T_0_ and subsequent generations (Milner et al., 2018). Crude DNA extractions were performed on leaf tissues sampled during the jiffy pellet growth stage.

### Mutation identification

Primers were designed to amplify each GSK1 region in the three species encompassing all three guide targets using the crude DNA extracts as above (**Supplementary Table 2**). PCRs were performed using Phusion Hot Start II High-Fidelity PCR Master Mix (Thermo Fisher Scientific) and T_m_ for each primer set were used based on the prediction using the online tool (https://www.thermofisher.com/uk/en/home/brands/thermo-scientific/molecular-biology/molecular-biology-learning-center/molecular-biology-resource-library/thermo-scientific-web-tools/tm-calculator.html). Sequence mutations were determined by Sanger sequencing. Typical chromatograms for WT and mutant OSGSK1 lines are shown in **Supplementary Figure 1**. Editing efficiency was calculated on a per plant basis; given the hexaploid genome in wheat, a wheat plant with an edit in any of the A, B or D homoeologues was considered edited. Data for individual wheat homoeologue-specific edits is also included for the individual guide and stacking strategies.

### RNA isolation and expression analysis

The leaf tissue from four independent transgenic rice, and wheat plants were flash frozen in liquid nitrogen. Total RNA was isolated using a RNeasy Kit (Spectrum plant total RNA kit from Merck) and treated with DNase I (Thermo Fisher Scientific Inc.) prior to cDNA synthesis from 2 μg of total RNA using Superscript IV RT Kit (Invitrogen). The cDNA was used as template for semi-quantitative PCR reaction. Transcripts of GUS and Luc were detected simultaneously using specific primers with Phire Green Hot Start II PCR Master Mix (Thermo Fisher Scientific Inc.). Cycle conditions included an initial denaturation step (2 min at 94°C) followed by 30 cycles (30 s at 94°C, 30 s at 61°C, and 45 s at 72°C) and a final elongation step (7 min at 72°C) allowing for semiquantitative analysis of each reaction. All four primers were added into each individual reaction. Amplicons (ranging from 208 to 554 bp) were resolved in 1% agarose gels. The luciferase gene served as a constitutively expressed transformation control for the normalization of GUS expression levels.

### Quantification of band intensities and statistical analysis

Band intensities were quantified using ImageJ software (Schindelin et al., 2012) to measure relative expression levels. The graph visually illustrates the relative expression patterns of GUS transcripts normalized to luciferase in rice and wheat for the three promoters analyzed. The error bars depict the standard error of the mean across four independent lines. Statistical significance calculated using Tukey’s Honest significance test in the GraphPad Prism software, with significance levels are denoted as follows: * for p < 0.05; ** for p < 0.01; 0.001; and **** for p < 0.0001.

### Chromatin state

Publicly available data sets from Concia et al., 2020; and Yuan et al., 2022 were downloaded and analyzed for their read depth at the known genomic locations in the Chinese Spring reference sequence. Publicly available expression data was downloaded from wheat-expression.com.

### T_1_ germination and assessment

T_1_ seeds from selected rice, wheat and barley lines were sown in Jiffy-7 pellets and germinated in a controlled environmental chamber as above with rice being grown at 28.5°C day/23.5°C night, 16 hour daylength. Barley at 18°C day/13°C night, 16 h daylength, and wheat at 20°C day/15°C night, 16 h daylength. NptII copy number and edit identification analyses were carried out as above. Significance was determined using Chi-square test on observed inheritance compared to the expected probabilities for Mendelian segregation.

## Results

To understand how to best achieve stable heritable editing in important crop species and to understand the role that guide sequence plays in the editing of cereals we tested three guides targeting the GSK1 kinase in rice, barley, and wheat. We wanted to identify guides which would target the same DNA sequence in the homologous genes in the three different species to allow direct comparisons to be made. Comparative analysis of protein homology revealed > 86% sequence identity of GSK1 among these three species. The most divergent is the predicted gene model for TaGSK1-D as the 5’ end of the gene has not been fully resolved in the reference sequence and the sequence appears to lack the first exon due to the lack of available public sequence. All three wheat gene models appear to be lacking at least the first exon. We also noticed that a gene model for *TaGSK1-B* is absent in wheat Refseq 1.1, but a strong BLAST hit does exist in the wheat genome on chromosome 3B. There is a gene model for *TaGSK1-B* in wheat using the Refseq2.1 gene models, but it is listed as a low confidence gene. Therefore, for comparative purposes we used the Refseq 2.1 predicted sequences for evaluation of the predicted amino acid and DNA coding sequences (**Supplementary Tables 3**, **4**). Common guides were designed to target each of the five loci, within the three species allowing for a direct comparison between guide delivery strategy and species. From this we chose three guides and used them in *Agrobacterium*-mediated stable transformation experiments to assess both the mutation rates as well as their inheritance in the T_1_ generation. Using a guide prediction system such as CRISPR-P 2.0 these guides were scored as 0.4148, 0.2378 and 0.3939 for guides 1, 2 and 3 respectively (Liu et al., 2017). A schematic diagram of the pMM36 and pMM37 T-DNAs are shown in **Figure 1** with locations of the three guide targets in rice, barley and the A-homoeologue of wheat.

### Rice

In order to compare the CmYLCV-expressed three-guide stack presented either by a tRNA system or a ribozyme system, 25 T_0_ plants containing the tRNA system and 32 T_0_ plants containing the ribozyme system were produced and confirmed to contain at least one T-DNA by qPCR Taqman assay. Using the tRNA system the likelihood of editing at any of the three targets in a single plant was 76% (19/25 plants) compared with 62.5% using the ribozyme system (20/32 plants) (**Supplementary Table 5**). Overall, the least efficient guide was guide 1 which produced edits in 52.0% or 31.3% of the plants containing the tRNA or ribozyme guide stack, respectively. Guide 2 produced edits in 63.6% (14/22 plants genotyped) or 55.6% (15/27 plants) respectively. The third guide was the most efficient with edits observed in 68% of plants (17/25 plants) using the tRNA versus 53.1% (17/32 plants) using the ribozyme system.

Six single-copy rice lines which showed editing in the T_0_ were selected and grown on to the next generation. This included three lines with a tRNA guide delivery system and three lines with a ribozyme guide delivery system. Sixteen plants of each line were then genotyped for both the T-DNA copy number and the mutations at the three guide locations. Analysis of the T_1_ plants showed that five out of the six lines tested had heritable edits identified in the T_0_ material. The line MR43.10, which originally showed a large deletion (1016 bp), did not inherit the identified mutation in the T0 plant but did show a new mutation in the T1 material at guide 2. In four of the six rice lines tested, new mutations were seen in the T_1_ progeny at each of the three guide locations (**Supplementary Table 6**).

### Barley

**Figure 3:**
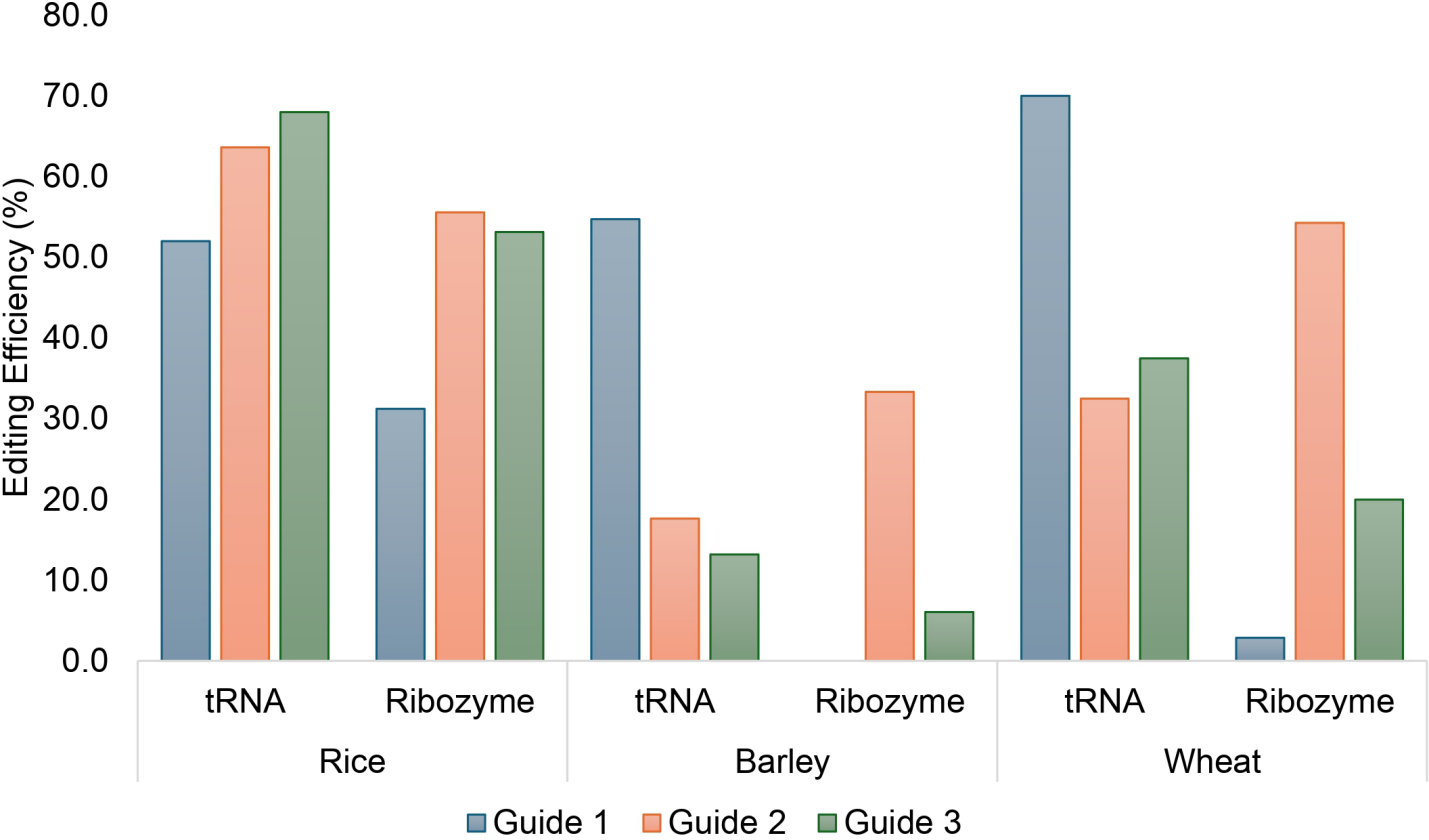
Comparison of CRISPR/Cas9-mediated editing efficiency of three guides targeting *GSK1* in rice, wheat and barley delivered as a tRNA or ribozyme system in T_0_ rice, barley and wheat plants.

Similarly, 53 and 33 transgenic barley plants were created containing either the tRNA or ribozyme guide delivery system. Overall, 54.7% of plants (29/53 plants) showed at least one edit at *HvGSK1* using the tRNA guide delivery system versus 33.3% (11/33 plants) using the ribozyme guide delivery system (**Supplementary Table 7**). We observed that with the tRNA system, guide 1 was the most active with 29 of the 53 edited plants mutated at the guide 1 site. Guide 2 and guide 3 edited plants had reduced but similar editing rates, with 9 and 7 plants respectively (**Figure 3**). The ribozyme system was less efficient than the tRNA guide delivery system at creating edits in barley. Editing in barley containing the ribozyme guide stack was mainly driven by guide 2 as all the plants that showed an edit were mutated at the guide 2 site. No plants showed an edit at the guide 1 site and guide 3 only edited the desired sequence in two of the 33 plants produced.

Inheritance of the mutations was again high as 85.7% of the mutations identified in the selected single copy lines showed a Mendelian pattern of inheritance in the T_1_ generation. Similar to the data observed in rice the only mutation not inherited was a large deletion observed in line Hv17B.2. Again, as seen in rice, new mutations were observed in the T_1_ material. These included seven new mutations in the three tRNA lines taken forward whereas no new edits were seen in the ribozyme lines. Many of the new edits observed in these lines did not show the expected segregation ratio of 1:2:1 suggesting that the mutation might have arisen in a germ line cell late in the life cycle (**Supplementary Table 8**). Unlike rice, mutations in barley could be identified in the T_1_ generation in lines where the T-DNA bearing Cas9 had been segregated out. This suggests that the activity of the Cas9 in the T_0_ generation was still producing new mutations which could potentially be inherited, and that inheritance of the previously identified mutations was not a given as previously observed (Howells et al., 2018). Again, this activity was only observed in two of the three tRNA lines taken forward suggesting high activity of the tRNA system in barley relative to the ribozyme system. This underscores the importance of confirming both the edit and its inheritance whilst quickly removing the T-DNA to achieve a stable genotype prior to any functional gene analysis, particularly in barley.

### Wheat

In wheat 40 and 35 plants were regenerated, with either the tRNA or ribozyme constructs to deliver the guides on the T-DNA. A high level of editing was seen in the T_0_ plants. Overall, 72.5% of plants showed at least one edit in any homoeologue and 47.5% of plants (19/40) contained edits at all three homoeologue sites with guide 1 being the most active guide (A-47.5%, B-47.5%, D-60.0%) followed by guide 3 (A-32.5%, B-20.0%, 10.0%) and finally guide 2 (A-9.4%, B-20.0%, D-20.5%) as the least efficient guide using the tRNA system (**Figure 3**; **Supplementary Table 9**).

In wheat the ribozyme system again showed relatively poor editing performance compared to the tRNA system with only 57.1% of plants (20/35) showing at least one edit at any homoeologue and only 5.7% of plants (2/35) showing edits at all three homoeologues. Again, as seen in barley a large difference was seen in the editing only editing one plant at the D homoeologue of *TaGSK1* (2.9%) and no edits were observed from guide 1 at either the A or B homoeologues. The ribozyme system showed that guide 2 was the most active with up to 45.7% (A-2.9%, B-45.7, D-40.0) of plants showing an edit at one of the homoeologous target sites. This was followed by guide 3 showing the highest editing of the B homoeologue at 17% (A-2.9%, B-17.1%, D-5.7%). There was a significant deviation in the expected editing efficiency between homoeologues using the ribozyme system as only 2 plants were edited at the A homoeologue compared with 16 for B and 14 for the D homoeologue (χ2 = 0.005). This was not observed with the tRNA system as 24 of A, 21 of B or 26 of D homoeologues were successfully edited (χ2 = 0.765).

The inheritance of the mutations in wheat was strong overall with only one of nineteen previously identified mutations not inherited in the T_1_ generation. Again, additional novel mutations were identified in the T_1_ plants suggesting that the Cas9 is active throughout the whole generation and segregation of the T-DNA is necessary to control for new edits. The new mutations were always observed at the most active guide site for both the tRNA and ribozyme systems in wheat. It should be noted that two of the mutations did not show typical inheritance patterns with each showing a 1:1 inheritance (**Supplementary Table 10**). This may suggest the original identified mutation was not inherited and the mutation was possibly created later in the life cycle, as seen in barley. In contrast to barley, where new mutations were seen in plants which retained the T-DNA in all six lines tested, wheat lines where the T-DNA had been segregated out contained new mutations. However, the tRNA system only showed 10 new mutations in the T_1_ plants including one in a line lacking a T-DNA, whereas 38 new mutations were seen in the T_1_ with nine plants showing mutations in plants which lacked a T-DNA suggesting again that these mutations happened later in the T_0_ life cycle.

### Promoter activity

**Figure 4:**
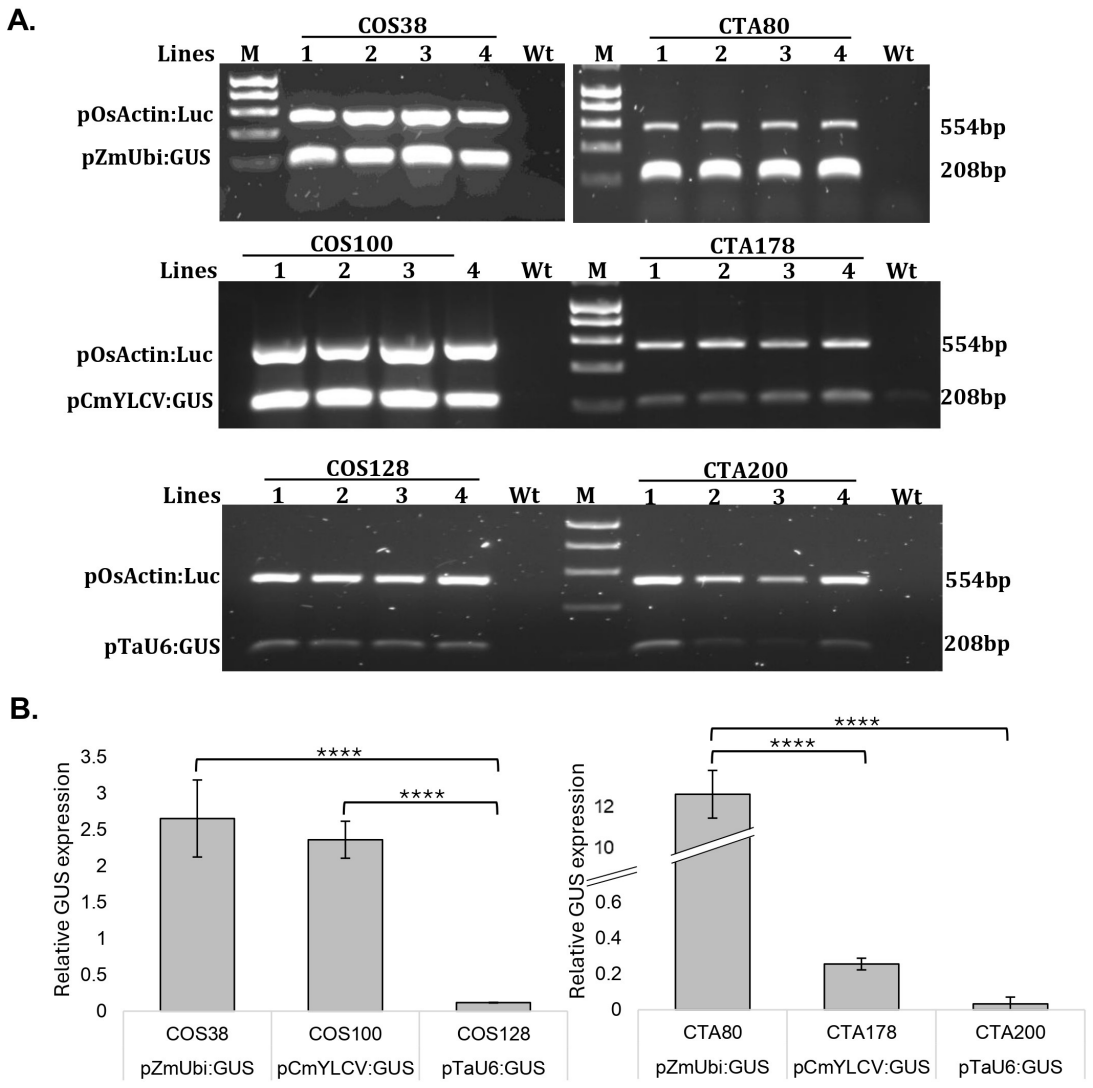
Relative GUS Expression Assessed by Semiquantitative PCR. Agarose gels showing OsActin: Luciferase and GUS transcript levels expressed from the pZmUbi, pCmYLCV and pTaU6 promoters in four independent rice (COS) and wheat (CTA) plants **(A)**. M-1Kb gel marker, Wt- non-transformed control plant. Agarose gel bands were quantified using Image J software, and the resulting values were normalized against the luciferase, which served as the transformation control. The fold change was then calculated against the luciferase reference, illustrating the relative expression levels of GUS **(B)**. The error bars depict the standard error of the mean across four independent lines. Utilizing Tukey’s Honest significance test, a notable difference is observed between pZmUbi and pTaU6 in both rice and wheat. In rice alone, a significant variance in promoter activity is evident between pCmYLCV and pTaU6. Significance levels are denoted as **** for p < 0.0001.

The expression profiles of the pCmYLCV: GUS, pTaU6:GUS, and pZmUbi: GUS in four independent transgenic rice and wheat lines were analyzed through semiquantitative PCR, coupled with the quantification of band intensity (**Figure 4**). To normalize the expression of each independent transformed line a comparison to the expression of the pOsActin: Luc cassette included in each T-DNA enabled assessment between stable transgenic lines independent of T-DNA insertion site(s).

Based on the experimental data, the CmYLCV promoter demonstrated a similar robust level of expression in rice compared with the constitutive ZmUbi promoter, but in wheat CmYLCV expression was markedly weaker compared with ZmUbi. Expression from the TaU6 promoter was notably lower in rice compared to both pZmUbi and pCmYLCV, with the difference observed to be statistically significant (p < 0.0001). Although CmYLCV exhibited higher expression in wheat than TaU6, this difference was not statistically significant. Notably, in rice, the CmYLCV promoter showed a fold difference of approximately 20X compared to TaU6, whereas in wheat, this difference was not as pronounced, with CmYLCV being only about 2X higher than TaU6. These results indicate that the CmYLCV promoter drives higher expression levels compared to the TaU6 promoter in both rice and wheat, with this difference being more pronounced in rice. Although these findings suggest that CmYLCV could be a more effective promoter for driving the expression of guide cassettes in gene editing experiments, this study did not directly evaluate the impact of these promoters on editing efficiency within the same guide delivery system. Therefore, while our observations are consistent with the higher editing efficiency associated with the ribozyme system as reported by Li et al. (2021), our results do not provide direct evidence that the CmYLCV promoter is superior to TaU6 in enhancing CRISPR/Cas9-mediated editing efficiency. Further studies would be required to confirm this potential advantage in the context of gene editing.

### Chromatin landscape

To understand why such a significant difference was seen between the editing efficiency of the individual wheat homoeologues when using the ribozyme system, we took publicly available ATAC sequence data to examine the accessibility of the various homoeologues to the CRISPR/Cas9 machinery (Concia et al., 2020; Yuan et al., 2022). The relative accessibility of the three homoeologues showed contrasting profiles. Locations for the B and D homoeologues show relatively deep levels of sequence suggesting an open stretch of chromatin. However, the homoeologue of *TaGSK1* on the A-genome shows relatively no sequencing depth suggesting that the DNA may be mainly in a heterochromatin state. Examination of the expression databases suggests that this does not change the expression patterns of the homoeologues as the A and D homoeologues are expressed at similar levels in the roots, shoots, and spike (**Supplementary Figure 2**).

## Discussion

We have focused on two key aspects of the CRISPR system – promoter selection and guide delivery strategy to understand how to best approach targeted mutagenesis in important cereal crop species such as rice, barley, and wheat. The data collected here and by others suggests that the CmYLCV promoter is highly effective for expression of transgene expression in these crop species, or applications where expression of non-coding RNAs, such as CRISPR guides, are required for effective gene editing strategies (Čermák et al., 2017; Li et al., 2021). We chose the CmYLCV promoter and identical guide sequences to test the effectiveness of two guide delivery systems to determine whether common components would be equally effective across different cereal species. In the three species tested, large differences were seen in the overall editing rate at any of the loci and the guides which were active more than others. In rice, both systems work reasonably well with more than 60% of the plants showing edits from either system. While the guide prediction program was not able to predict accurately which guide location would be most efficiently edited, differences in the editing at a given guide location were observed between the two systems. This has been observed previously in a few species, but here we present further evidence in multiple species that some of the guide prediction programs are good for identification of guides but are poor in predicting the actual editing rates in the plant (Milner et al., 2020b; Naim et al., 2020). Despite this limitation multiple examples were seen in each species where larger deletions of the intervening GSK1 gene sequence between two or more guide target sites were identified.

Analysis of the two guide delivery systems in barley shows that the tRNA system is by far superior to the ribozyme delivery system. For example, guide 1 produced edits in 54.7% of the analyzed T_0_ barley plants with the tRNA system compared with 0% of T_0_ plants from the ribozyme system despite guide 2- and 3-mediated edits being introduced by the ribozyme strategy. Guide 2 was the most effective in targeting edits in 33.3% of T_0_ plants when using the ribozyme system. This is similar to previous observations in barley protoplasts where the tRNA system performed better than the ribozyme (Čermák et al., 2017).

In wheat, the results mirrored those from barley, with the tRNA system far more efficient in generating edits than the ribozyme system. Wheat and barley also had similar favored guide profiles – with guide 1 in the tRNA system or guide 2 in the ribozyme system the most efficient at editing. The overall rate of editing was far higher in wheat using the tRNA system compared to the ribozyme system with 72.5% of the plants showing edits when using the tRNA system but only 57.1% when using the ribozyme system. This is crucial in a polyploid species such as wheat and almost an 8-fold increase in editing of all three homologues using the tRNA system (47.5%) compared with the ribozyme system (5.7%) was observed. This contrasts with previous reports where the ribozyme system showed the highest editing efficiency (Li et al., 2021). However, this work demonstrates that the promoter selected for guide expression matters; pol III promoters are generally thought to provide lower transcript levels than CmYLCV and therefore promote lower editing efficiency (Čermák et al., 2017; Li et al., 2021). This work also clearly shows that lower expression of transcript is seen with TaU6 than the CmYLCV promoter in rice and the same general trend is seen in wheat (**Figure 4**). Surprisingly, large differences were also observed in the editing of homoeologues when using the ribozyme system but not the tRNA system. This might be due to the accessibility of Cas9 complex to DNA as the chromatin state has been suggested to alter editing ability (Weiss et al., 2022). If this hypothesis held true, we would expect to observe differences in the editing of homoeologues for both the tRNA and ribozyme systems which was not the case. This indicates that accessibility alone cannot explain variations observed in homoeologue editing, suggesting other factors may also play an important role.

One of the key findings of this work is that the guide sequences themselves may be less important in editing efficiency *per se* than the manner in which they were presented. In rice both strategies broadly worked very well, and all three guides showed comparable editing efficiencies in each guide system. However, in wheat and barley, the choice of guide system had a large influence on the editing efficiency, with editing rates for any particular guide varying by over 50% in barley and 40% in wheat. This was unexpected as again both guide delivery systems have been shown to work in barley and wheat (Čermák et al., 2017; Li et al., 2021). There also seemed to be no consistency in the effectiveness of a particular guide sequence compared with other guides in the guide stack. For instance, in rice, guides 2 and 3 work at similar rates in both the tRNA and ribozyme systems but in barley and wheat, guide 1 was the best for the tRNA system, whereas guide 2 was the best for the ribozyme system. Pre-screening for efficient guides in advance could be useful if transient delivery systems are established. In some instances, a PCR/restriction digest can be used to identify mutant amplicons (Shan et al., 2014). This is cost-effective but for other targets a suitable restriction site may not be available in the wild type sequence and deep sequencing of amplicons is required. Effort and cost of the procedure therefore need to be balanced and at a practical level, researchers currently work around this limitation by stacking two or three guides per target gene. Factors outside of the sequence itself may also need to be considered such as cellular pH, ionic strength, and temperature, which could influence the ribozyme conformational structure and autocatalytic cleavage efficiency. One fundamental difference between the cereal transformation systems used here is the use of mature seed-derived callus as the tissue source for *Agrobacterium*-mediated rice transformation in contrast to freshly harvested immature seed for wheat and barley transformation. It might be anticipated that dedifferentiated actively dividing callus cells may respond differently compared with relatively quiescent immature embryo cells or that differential transcript/guide expression or post transcriptional RNA processing might occur. This hypothesis could be tested by transforming immature rice embryo and mature seed callus with identical gene editing constructs.

In our experiments rice-Agrobacterium co-cultivation and tissue culture stages plus the callus regeneration stages of the wheat and barley tissue culture were all performed at 28°C, to maximise expression of Cas9 from the ZmUbi promoter and promote early editing (Milner et al., 2020b). It therefore appears unlikely that temperature *per se* is responsible for this difference in ribozyme activity between rice compared with wheat and barley in these crucial early stages. Nevertheless, further gains might be achieved by elevating the temperature for the wheat and barley callus-induction stages to 37°C (Milner et al., 2020b). Other factors such as cellular pH or ionic strength may be more difficult to approach, although manipulation of the ribozyme structure to optimize efficacy within a particular cell type might warrant further investigation.

One final point that needs highlighting is the relative expression differences of the guide RNAs driven by the same promoters in the different species. While we cannot make a direct comparison of strength of promoters between species, large differences were observed in relative promoter strengths between rice and wheat in the three promoters tested (**Figure 4**). While in rice the expression of the guide RNA and Cas9 were relatively equal, in wheat more than 20X higher transcript levels are expected for the Cas9 relative to the gRNA. After measuring the strength of the two promoters used in the study to drive the Cas9 and guide RNAs other promoters such as OsActin might be better for driving the guide RNA expression other than either the polIII promoter TaU6 or CmYLCV which are more common in the literature (Shan et al., 2014; Čermák et al., 2017; Howells et al., 2018; Hahn et al., 2020; Milner et al., 2020a; Li et al., 2021). This seems to be more important in wheat than rice as CmYLCV was a fraction of the expression of the ZmUbi promoter used to drive the Cas9 in wheat but nearly identical in rice. This result was surprising as previous studies have shown that CmYLCV has strong expression in a number of species including monocots such as maize and rice (Stavolone et al., 2003). This variability across species underscores the need to match promoter strength with the cellular environment to optimize editing outcomes in cereal gene editing.

In summary, this work highlights the significant impact of the chosen guide delivery system on the success of the targeted editing. This work also demonstrates the variability among species in editing efficiently and emphasizes the importance of carefully considering and evaluating available tools for efficient editing in each individual species. Despite differences, both guide delivery systems were able to edit most targeted sequences in all three species. However, the tRNA system consistently outperformed the ribozyme system in all three species. While both systems showed similar efficiency in rice, notable differences were evident in wheat and barley. The high efficiency of the tRNA system in polyploid wheat demonstrates its suitability for species with complex genomes. Achieving robust editing across multiple homoeologous gene copies is essential in polyploid crops to generate complete knockouts or functional changes, especially for traits governed by redundant gene functions. Similarly, the use of a highly efficient tRNA system may also assist in successfully targeting multiple unrelated genes. The choice of system may be inconsequential for simple knock-out experiments employing multiple guides to create the desired mutations. However, for more precise editing tasks, such as prime editing, base editing, or allele replacement, the editing efficiency at any given location could significantly influence the experiment’s feasibility.

## Data Availability

The original contributions presented in the study are included in the article/**Supplementary Material**. Further inquiries can be directed to the corresponding author.
