# Supplementary figures and images for: Differential editing efficiencies in cereal crops: a comparative analysis of tRNA and ribozyme multiplexed guide delivery
Source: Front Plant Sci. 2024 Dec 5;15:1426184. doi: 10.3389/fpls.2024.1426184 (PMC11657133; doi:10.3389/fpls.2024.1426184)

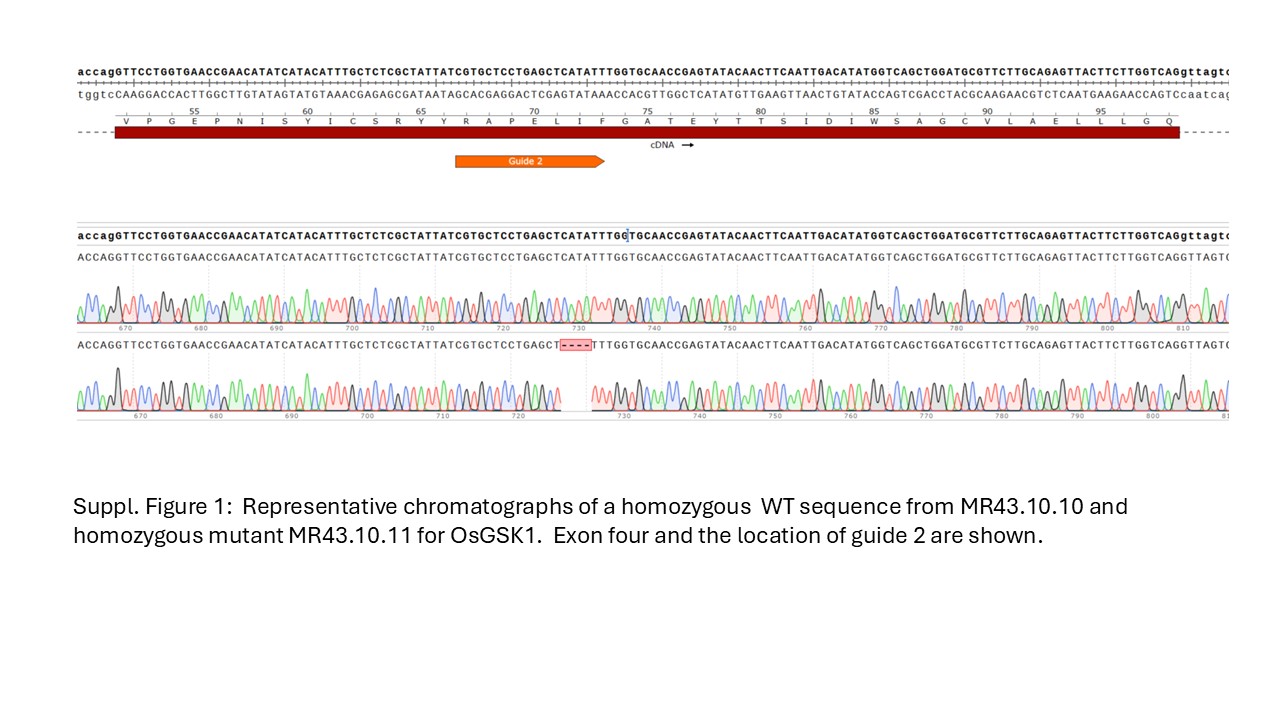

Supplement: Supplementary file 1 [file Image1.jpeg]

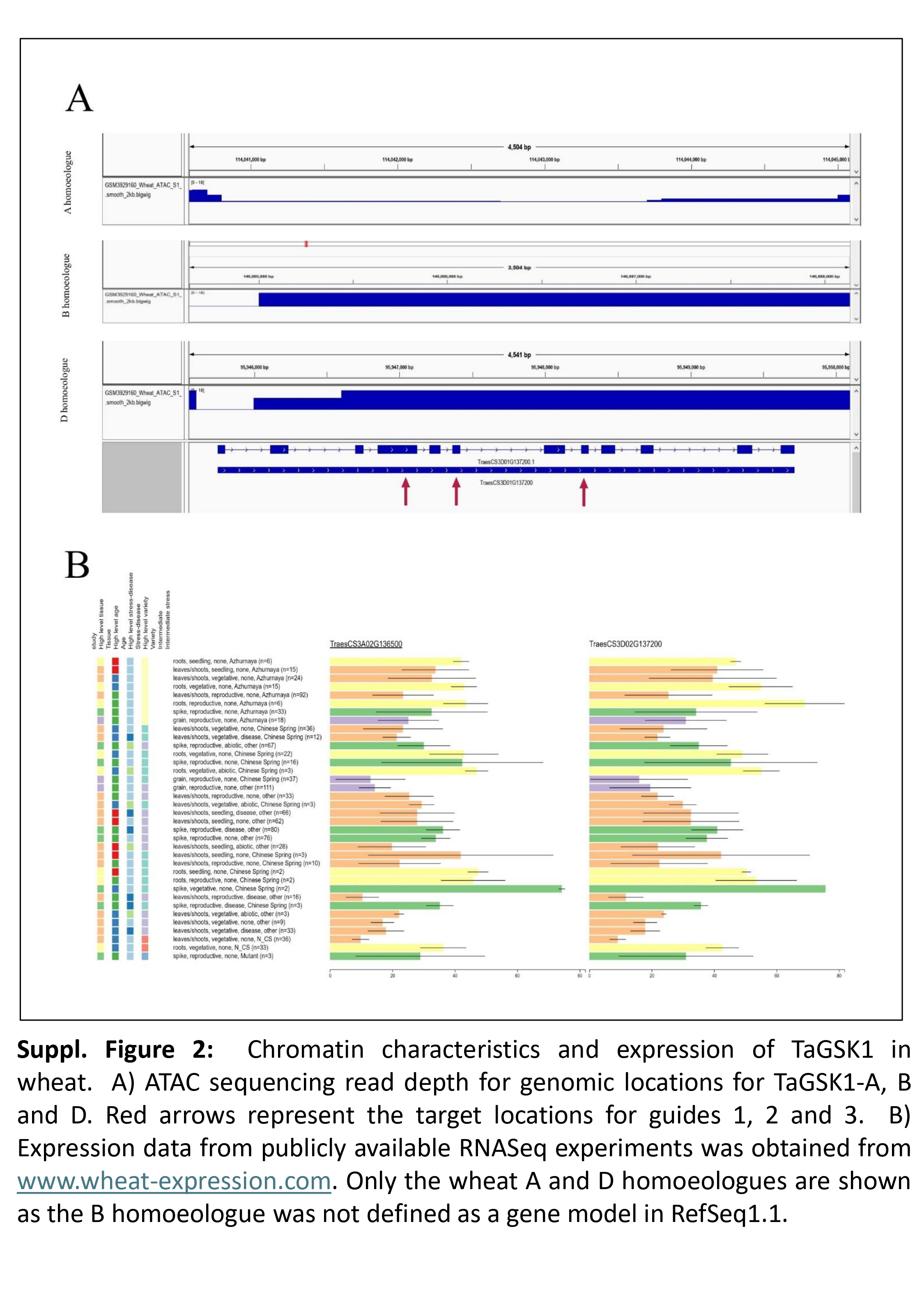

Supplement: Supplementary file 2 [file Image2.jpg]
